# Multi-Time Scale Trading Simulation of Source Grid Load Storage Based on Continuous Trading Mechanism for China

**DOI:** 10.3390/s22062363

**Published:** 2022-03-18

**Authors:** Xun Dou, Li Song, Shengnan Zhang, Lulu Ding, Ping Shao, Xiaojun Cao

**Affiliations:** 1 College of Electrical Engineering and Control Science, Nanjing TECH University, Nanjing 211816, China; xxxx_ding@163.com; 2 Beijing Electric Power Exchange Center, Beijing 100053, China; li-song@sgcc.com.cn (L.S.); shengnan-zhang@sgcc.com.cn (S.Z.); 3 China Electric Power Research Institute (Nanjing), Nanjing 210003, China; shao.ping@epri.sgcc.com.cn (P.S.); cao.xiaojun@epri.sgcc.com.cn (X.C.)

**Keywords:** source grid load storage, continuous trading mechanism, multi-time scale, trading simulation, interactive trading

## Abstract

The proportion of new energy in power systems is increasing yearly. How to deal with the adverse impact of new energy output uncertainty on its participation in trading from the mechanism level is an urgent problem in China that must be solved. A source grid load storage (SGLS) continuous trading mechanism and a multi-time scale trading simulation method are proposed which meet the needs of Chinese new energy consumption and satisfies the trading needs of Chinese power market players. Firstly, the connection mechanism of mid-long term, day-ahead, and intra-day SGLS interactive trading is established, and the meaning and ways of continuous development are defined. Secondly, the clearing model of SGLS trading based on the continuous trading mechanism is established to provide mathematical models and strategic methods for various resources to participate in SGLS trading. Then, the multi-time scale trading simulation of SGLS based on the continuous trading mechanism is carried out to obtain the trading strategies of different trading subjects. The example results show that compared with the trading mechanism based on deviation assessment, the one-day trading cost is reduced by 4.20% and the consumption rate of new energy is increased by 6.53%. It can be seen that the mid-long term–day-ahead–day SGLS interactive trading connection mechanism has advantages in reducing trading costs and improving the consumption rate of new energy. It can flexibly deal with the trading scenario of domestic new energy consumption and new energy reverse peak shaving, which has an effect on the adverse impact of trading and operation deviation caused by source load uncertainty on trading.

## 1. Introduction

**Figure 1:**
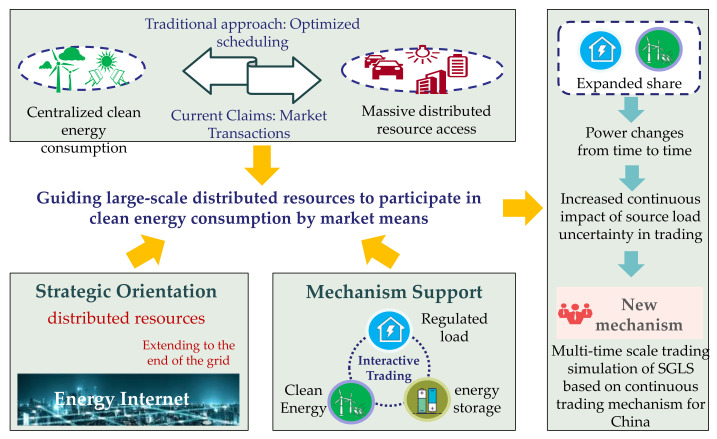
Background on the application of the continuous trading mechanism and simulation method for SGLS.

With the advancement of China power market reform, the construction of the power spot market continues to advance. Different from the mid-long term market, the spot market responds to more uncertain factors [1,2]. Especially under the dual carbon strategy, the proportion of new energy sources, mainly wind power and photovoltaic, continues to increase, improving the uncertainty of source side resources [3]. At present, the access of a large number of source-load resources has a large uncertainty and will have a large impact on the power grid. China’s power market is in the initial development stage, and the interface between the mid-long term market and the spot market is rather ambiguous and not enough to address the significant impact of source-side resource uncertainty. In order to ensure the orderly development of China’s power market trading, trading is carried out by using regulated load (RL) and energy storage (ES) resources to reduce the adverse impact of new energy uncertainty on trading, which has become one of the important research directions [4]. The market of SGLS is a transitional link from the mid-long term power market to the spot power market that is currently being prepared in China. Compared with the energy market and auxiliary service market, the trading behavior of different subjects of regional source, load and storage is considered in the market of SGLS, and independent clearing is carried out according to demand. It can be used to form a balance of energy supply and demand by market-based means. The SGLS trading involves mid-long term, day ahead, and intra-day time scales. However, due to the expanding share of domestic new energy and regulated load users, while their power uncertainty is changing from time to time [5], especially in the trading of SGLS across mid-long term, day ahead, and intra-day time scales, the continuous impact of source load uncertainty is greater. Therefore, it is necessary to carry out mid-long term, day-ahead, and intra-day continuous trading of SGLS to deal with the impact of source load uncertainty on the trading, as is shown in Figure 1.

The SGLS market runs through the mid-long term market and the spot market, taking into account new energy, grid, and load users, which can effectively consume surplus power and effectively connect the mid-long term market and the spot market. It can also provide a strong foundation for the development of China’s power market. However, there is less research involved in the SGLS market in China.

In terms of trading mechanism, a day-ahead trading market operation model suitable for China’s national conditions was proposed in [6], the corresponding organization process and operation rules were given, and a zonal tariff mechanism for the problem of network congestion or line cross-section tide crossing limits were proposed. In [7], a market operation mechanism for electricity auxiliary services was established, and a detailed description of its trading process, demand forecast and release, data declaration, bidding rules, trading mechanism, and scheduling of generation and transmission auxiliary services were provided. In [8], a virtual power plant optimization and scheduling method for electricity and heat interconnection considering the market trading mechanism was established, which can effectively solve the operation and market trading problems of complex energy systems including electricity, heat, natural gas, and other energy sources. In [9], through an in-depth study of the internal logic of the structure and institutional mechanism of the Brazilian power market, combined with the current situation of China’s power industry and the very different characteristics of the resource endowment of each province and region, insights and suggestions for the construction program and mechanism design of the electricity market from the perspective of promoting sustainable energy development and optimal utilization of energy resources were put forward. In [10], a power trading portfolio model for power generating companies considering the uncertainty in the electricity, fuel and carbon emission markets was constructed. In [11], a power market trading index system for power generation enterprises by considering four aspects, power trading price, market activity, settlement timeliness, and market efficiency, was established. However, these mechanisms mainly discuss how new energy can participate in the market trading, and are less concerned with ES, RL, and new energy coordination in trading.

For the trading of RL, a demand response trading mechanism considering the difference in response rate and formulated dynamic reward and punishment rules based on the principle of high quality and price was proposed in [12]. The research status and prospect of energy trading mode and user response of energy internet was discussed in [13]. In view of the irrationality of ES demand and resource allocation, a peer-to-peer trading mechanism for renewable energy systems with integrated hydrogen vehicle energy storage based on actual energy consumption and simulation data was established in [14]. Considering the business model of joint frequency regulation of shared ES and thermal power, a decentralized joint frequency regulation trading mechanism considering the preferences of distributed ES and thermal power units based on block chain technology was proposed in [15]. A distributed energy trading management approach for renewable energy generators and consumers was provided by combining fresh air systems (HVAC) with energy storage, which can effectively reduce the energy costs of users in [16]. In [17], a master-slave game trading method based on coordinated control of wind, storage, and electric boiler loads was proposed to exploit the regulable load potential using market incentives. The above mechanism mainly discussed how new energy, RL, and ES participated in trading, respectively, and rarely involves the coordinated participation of ES, RL, and new energy in trading.

In terms of trading strategy, a point-to-point (P2P) trading strategy for cloud ES with semi-distributed structured topology which provided theoretical support for user-side ES was proposed in [18]. In [19], a three-stage combined nuclear–thermal–virtual power plant peaking strategy considering a carbon trading mechanism was proposed, which can effectively reduce the cost of combined peaking. Based on the Cournot oligarch game, a local energy trading strategy for energy suppliers which can realize the local supply and demand balance between distributed generation and users was constructed in [20]. An aggregation trading strategy of SGLS was proposed in [21], it was mainly oriented to electric heating load. In [22], a peak regulation strategy of SGLS under different PV permeability was established, and an aggregation trading strategy of multi-energy load storage resources for a virtual power plant was proposed in [23]. In [24], a multi-energy clearing model with integrated energy service providers as the main market player was proposed. However, the current trading mechanism rarely involves the connection of mid-long term, day-ahead, and intra-day in a continuous time scale. Most of them study the trading of single time scale, it is difficult to deal with the uncertainty of new energy on mid-long term, day-ahead, and intra-day in a continuous time scale.

In view of the above problems, considering new energy uncertainty and the development demand of the SGLS interactive trading market, this paper proposes a continuous trading mechanism of SGLS that meets the consumption needs of new energy, meeting the trading needs of market subjects. It can fully compete and is relatively fair. A trading clearing model of SGLS based on the continuous trading mechanism is established. Multi-time scale trading simulation of SGLS based on the continuous trading mechanism is designed to obtain the trading strategies of different trading subjects.

The main innovations of this paper are as follows:

- Considering the uncertainty and regulated ability of SGLS, the connection mechanism of mid-long term, day-ahead, and intra-day SGLS interactive trading is built, and the connection relationship of monthly trading, day-ahead plan and intra-day rolling is established, it is conducive to reducing the impact of source load prediction uncertainty on trading and operation deviation.
- Considering the response characteristics and capacity of new energy, PL, and ES, a trading clearing model of SGLS based on the continuous trading mechanism is established, mathematical models and strategic methods for various resources are provided to participate in trading of SGLS, it can effectively reduce trading costs and improve the consumption rate of new energy.
- A multi-time scale trading simulation method of SGLS based on the continuous trading mechanism is constructed to provide mid-long term, day-ahead, and intra-day trading and monthly settlement simulation of SGLS, the multi-time scale trading simulation is realized, and a strategic scheme for the simulation of power spot trading is provided.

The organizational structure of this paper is as follows. The interactive trading connection mechanism of monthly, day-ahead, and intra-day in SGLS is built in Section 2, and the meaning and ways of continuous development are defined. In Section 3, the clearing model in trading of SGLS based on the continuous trading mechanism is established, mathematical models and strategies for various resources are provided to participate in trading of SGLS. In Section 4, the effectiveness of the proposed trading mechanism through an example analysis is verified, and the sensitivity of the trading strategy is analyzed through the comparison of trading schemes in different trading scenarios.

## 2. Interactive Trading Connection Mechanism of Mid-Long Term, Day-Ahead, Intra-Day in SGLS

**Figure 2:**
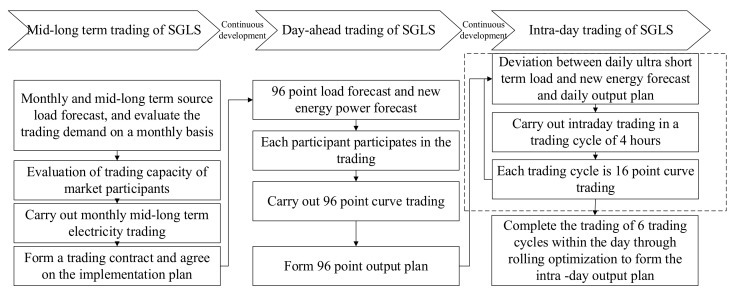
Continuous trading mechanism of monthly, day-ahead, and intra-day in SGLS.

For mid-long term trading, contracts can be signed according to the monthly source load forecast results, and the day-ahead trading can meet the needs of load users according to the day-ahead load forecast results. However, due to the continuity of new energy output and load change, there is still a large deviation between the day-ahead load forecast and new energy forecast after entering the intra-day time scale. Moreover, due to the output fluctuation of new energy under extreme weather and the load fluctuation on special holidays, the load demand of users on the current day, and the smoothing and consumption demand of new energy cannot be met only through day-ahead trading. Therefore, a continuous trading mechanism of SGLS from month to day is constructed. The market of SGLS is different from the spot market. It does not conduct trading matching through a third-party entity. Only different entities of regional internal load storage are considered to be cleared uniformly according to their needs. The specific connection method is shown in Figure 2. Firstly, the monthly mid-long term trading of SGLS is entered, the trading demand through the mid-long term source load forecast is evaluated, the mid-long term power trading with a monthly cycle is carried out, and the implementation scheme agreed in the trading contract is formed. Then, the day-ahead trading of SGLS is entered, each participant participates in the trading based on the 96 point source load forecast, and a 96 point output plan is generated through the 96 point curve trading. Then, the intra-day trading of SGLS is entered, intra-day trading in a 4 h trading cycle is conducted, 16 point curve trading clearing is carried out, the trading is completed in 6 trading cycles within the day through rolling optimization, an intra-day output plan is formed, and the deviation between trading and operation caused by the uncertainty of intra-day load prediction is reduced.

## 3. Trading Clearing Model of SGLS Based on Continuous Trading Mechanism

### 3.1. Monthly Trading Clearing Model of SGLS

#### 3.1.1. Objective Function

At the monthly time scale, the trading of SGLS is carried out. Combined with the trading rules of the existing mid-long term power market, the supply curve of electricity–price is submitted for new energy, ES and RL, and the demand curve of electricity–price is submitted for rigid load, as follows:

(1) $$\begin{array}{ll} \max C_{M} & =\sum_{i}\rho_{\mathrm{wtm},i}q_{\mathrm{wtm},i}+\sum_{j}\rho_{\mathrm{pvm},j}q_{\mathrm{pvm},j}\\ & +\sum_{k}\rho_{\mathrm{esm},k}q_{\mathrm{esm},k}+\sum_{m}\rho_{\mathrm{gem},m}q_{\mathrm{gem},m}\\ & +\sum_{n}\rho_{\mathrm{dlm},n}q_{\mathrm{dlm},n}-\sum_{l}\rho_{\mathrm{elm},l}q_{\mathrm{elm},l} \end{array}$$

where, *C*_M_ is the social welfare of monthly trading. *ρ*_wtm,*i*_ is the monthly quotation of the *i*th wind turbine(WT), *q*_wtm,*i*_ is the monthly volume report of the *i*th WT, *ρ*_pvm,*j*_ is the monthly quotation of the *j*th photovoltaic(PV) unit, *q*_pvm,*j*_ is the monthly volume report of the *j*th PV unit, *ρ*_esm,*k*_ is the monthly quotation of the *k*th ES, *q*_esm,*k*_ is the monthly report of the *k*th ES, and *ρ*_gem,*m*_ is the monthly quotation of the *m*th thermal power unit, *q*_gem,*m*_ is the monthly volume report of the *m*th thermal power unit, *ρ*_dlm,*n*_ is the monthly quotation of the *n*th RL, *q*_dlm,*n*_ is the monthly report of the *n*th RL, *ρ*_elm,*l*,*t*_ is the monthly quotation of the *l*th load user, and *q*_elm,*l*,*t*_ is the monthly report of the *l*th load user.

#### 3.1.2. Constraint Condition

The constraints to be met for the monthly trading of SGLS include the constraints of supply and demand balance and the trading constraints of various resources. The specific constraints are as follows:

1. Supply and demand balance constraints

For monthly mid-long term trading, the balance between supply curve and demand curve should be ensured at the time of trading, as follows:

(2) $$\begin{array}{l} \sum_{l}q_{\mathrm{elm},l}-\sum_{n}q_{\mathrm{dlm},n}\\ =\sum_{i}q_{\mathrm{wtm},i}+\sum_{j}q_{\mathrm{pvm},j}\\ +\sum_{k}q_{\mathrm{esm},k}+\sum_{m}q_{\mathrm{gem},m} \end{array}$$

2. Trading constraints of WT and PV renewable energy

WT and PV analyzed the maximum supply power and minimum supply power based on its own monthly power forecast, and an offer function is established, it is ex-pressed according to the market law and cost function as follows:

(3) $$\rho_{\mathrm{wtm},i}=a_{\mathrm{wtm},i}q_{\mathrm{wtm},i}+b_{\mathrm{wtm},i}$$

(4) $$\rho_{\mathrm{pvm},j}=a_{\mathrm{pvm},j}q_{\mathrm{pvm},j}+b_{\mathrm{pvm},j}$$

(5) $$p_{\mathrm{wtm},i,\min}\leq q_{\mathrm{wtm},i,t}\leq p_{\mathrm{wtm},i,\max}$$

(6) $$p_{\mathrm{pvm},j,\min}\leq q_{\mathrm{pvm},j,t}\leq p_{\mathrm{pvm},j,\max}$$

where, *a*_wtm,*i*_ is the primary term coefficient of the *i*th WT in the monthly quotation, *b*_wtm,*i*_ is quotation constant term coefficient of the *i*th WT in the month, *a*_pvm,*j*_ is the primary term coefficient of the quotation of the *j*th PV unit in the month, *b*_pvm,*j*_ is the coefficient of quotation constant term of the *j*th PV unit in the month, *p*_wtm,*i*,max_ and *p*_wtm,*i*,min_ are the upper and lower limits of monthly predicted power generation of the *i*th WT, respectively, and *p*_pvm,*i*,max_ and *p*_pvm,*i*,min_ are the upper and lower limits of monthly predicted power generation of the *j*th PV unit, respectively.

3. Trading constraints of ES

According to its own charging and discharging mode, the ES predicts the monthly discharge, analyzes the maximum supply and minimum supply, and a quotation function is established. According to the market law and cost function, it is specifically expressed as follows:

(7) $$\rho_{\mathrm{esm},k}=a_{\mathrm{esm},k}q_{\mathrm{esm},k}+b_{\mathrm{esm},k}$$

(8) $$p_{\mathrm{esm},k,\min}\leq q_{\mathrm{esm},k}\leq p_{\mathrm{esm},k,\max}$$

where, *a*_esm,*k*_ is the once term coefficient of monthly quotation for the *k*th ES, *b*_esm,*k*_ is the coefficient of quotation constant term of the *k*th ES in the month, *p*_esm,*k*,max_ and *p*_esm,*k*,min_ are the upper and lower limits of monthly predicted power generation of the *k*th ES, respectively.

4. Trading constraints of thermal power units

Thermal units are likewise forecasted to have upper and lower limits of generation in a month based on their characteristics, and offer functions are established as follows:

(9) $$\rho_{\mathrm{gem},m}=a_{\mathrm{gem},m}q_{\mathrm{gem},m}+b_{\mathrm{gem},m}$$

(10) $$p_{\mathrm{gem},m,\min}\leq q_{\mathrm{gem},m}\leq p_{\mathrm{gem},m,\max}$$

where, *a*_gem,*m*_ and *b*_gem,*m*_ are the primary and constant term coefficients of the monthly offer function for the *m*th thermal unit, respectively. *p*_gem,*m*,max_ and *p*_gem,*m*,min_ are the upper and lower limits of the forecasted generation of the *m*th thermal unit in the month, respectively.

5. Trading constraints with RL

The upper and lower limits of RL are determined according to the monthly trading volume forecast, and the quotation function is established, as follows:

(11) $$\rho_{\mathrm{dlm},n}=a_{\mathrm{dlm},n}q_{\mathrm{dlm},n}+b_{\mathrm{dlm},n}$$

(12) $$p_{\mathrm{dlm},n,\min}\leq q_{\mathrm{dlm},n}\leq p_{\mathrm{dlm},n,\max}$$

where, *q*_dl,*n*,max_ was the upper power limit of the *n*th RL, *q*_dlm,*n*_ is the upper limit of all-day regulated power for the *n*th RL, *a*_dlm,*n*_ and *b*_dlm,*n*_ are the primary term coefficient and constant term coefficient of the monthly offer function for the *n*th RL, respectively, and *p*_dlm,*n*,max_ and *p*_dlm,*n*,min_ are the upper and lower limits of the forecasted trading volume for the *n*th RL in a month, respectively.

### 3.2. Day-Ahead Trading Clearing Model of SGLS

#### 3.2.1. Objective Function

On the day-ahead time scale, 96-point trading of SGLS with curves are carried out, combining the trading rules of the existing day-ahead market, with ES and RL declaring 96-point tariff curves and power curves, and rigid load declaring 96-point power curves, as shown below:

(13) $$\begin{array}{ll} \min C_{\mathrm{DA}} & =\sum_{t\in T}(\sum_{i}\rho_{\mathrm{wtda},i,t}q_{\mathrm{wtda},i,t}\\ & +\sum_{j}\rho_{\mathrm{pvda},j,t}q_{\mathrm{pvda},j,t}\\ & +\sum_{k}\rho_{\mathrm{esda},k,t}q_{\mathrm{esda},k,t}\\ & +\sum_{m}\rho_{\mathrm{geda},m,t}q_{\mathrm{geda},m,t}\\ & +\sum_{n}\rho_{\mathrm{dlda},n,t}q_{\mathrm{dlda},n,t}) \end{array}$$

where, *C*_DA_ is the total cost of day-ahead trading. *ρ*_wtda,*i*,*t*_ is quotation of the *i*th WT in the day-ahead *t* period, *q*_wtda,*i*,*t*_ is the winning power of the *i*th WT in day-ahead time slot *t*, *ρ*_pvda,*j*,*t*_ is the offer of the *j*th WT and PV in day-ahead time slot *t*, *q*_pvda,*j*,*t*_ is the winning power of the *j*th WT and PV in day-ahead time slot *t*, *ρ*_esda,*k*,*t*_ is the offer of the *k*th ES in day-ahead time slot *t*, *q*_esda,*k*,*t*_ is the winning power of the *k*th ES in day-ahead time slot *t*, and *ρ*_geda,*m*,*t*_ is the offer for the *m*th thermal unit in day-ahead time period *t*, *q*_geda,*m*,*t*_ is the winning power for the *m*th thermal unit in day-ahead time period *t*, *ρ*_dlda,*n*,*t*_ is the offer for the *n*th RL in day-ahead time period *t*, and *q*_dlda,*n*,*t*_ is the winning power for the *n*th RL in day-ahead time period *t*.

#### 3.2.2. Constraint Condition

The constraints to be met by the day-ahead trading of SGLS include supply and demand balance constraints and trading constraints for each type of resource, as shown below:

1. Supply and demand balance constraints

The supply and demand balance constraints to be met by the integrated trading of WT, PV, ES, and RL resources to achieve a balance between supply and resources demand of SGLS are shown as follows:

(14) $$\begin{array}{l} \sum_{l}q_{\mathrm{elda},l,t}-\sum_{n}q_{\mathrm{dlda},n,t}\\ =\sum_{i}q_{\mathrm{wtda},i,t}+\sum_{j}q_{\mathrm{pvda},j,t}\\ +\sum_{k}q_{\mathrm{esda},k,t}+\sum_{m}q_{\mathrm{geda},m,t} \end{array}$$

where, *q*_elda,*l*,*t*_ is the load of the *l*th load user in the time period *t* before the day.

2. Trading constraints of WT and PV renewable energy

Based on the renewable energy trading currently conducted in China, the quoted prices for WT and PV are closely related to investment O&M costs and subsidies and are set to be constant during the trading cycle. The volume of WT and PV would not exceed their maximum output, as follows:

(15) $$q_{\mathrm{wtda},i,t}\leq p_{\mathrm{wtda},i,t}$$

(16) $$q_{\mathrm{pvda},j,t}\leq p_{\mathrm{pvda},j,t}$$

where, *p*_wtda,*i*,*t*_ is the predicted power of the *i*th WT in the time period *t* before the day, and *p*_pvda,*i*,*t*_ is the predicted power of the *j*th WT and PV in the time period *t* before the day.

3. Trading constraints of energy storage

The ES is profitable through the price of the trading declaration discharge, and it is set to a constant offer during the trading cycle, it is subject to its own operational constraints during the trading process, as follows:

(17) $$q_{\mathrm{esda},k,t}=p_{\mathrm{dis},k,t}$$

(18) $$E_{\mathrm{es},k,t}=E_{\mathrm{es},k,t-1}+p_{\mathrm{ch},k,t}\eta_{\mathrm{ch}}\Delta t-\frac{p_{\mathrm{dis},k,t}\Delta t}{\eta_{\mathrm{dis}}}$$

(19) $$0.2E_{\mathrm{esr},k}\leq E_{\mathrm{es},k,t}\leq 0.8E_{\mathrm{esr},k}$$

(20) $$E_{\mathrm{es},k,1}=E_{\mathrm{es},k,T}$$

(21) $$0\leq p_{\mathrm{ch},k,t}\leq p_{\mathrm{ch},k,\max}$$

(22) $$0\leq P_{\mathrm{dis},k,t}\leq P_{\mathrm{dis},k,\max}$$

where, *E*_es,*k*,*t*_ is the remaining capacity of the *k*th ES in the time period before day *t*, *p*_ch,*k*,*t*_ and *p*_dis,*k*,*t*_ are the charging and discharging powers of the *k*th ES in the time period before day *t*, respectively, *η*_ch_ and *η*_dis_ are the charging and discharging efficiencies of the ES, respectively, *E*_esr,*k*_ is the maximum capacity of the *k*th ES, and *p*_ch,*k*,max_ and *p*_dis,*k*,max_ are the charging and discharging power limits of the *k*th ES, respectively.

4. Trading constraints of thermal power units

The cost of thermal power units is closely related to their generation power, and the quoted price in the trading cycle is quadratically related to their trading power, and the trading process needs to meet its own operational constraints, as follows:

(23) $$\rho_{\mathrm{geda},m,t}=a_{\mathrm{geda},m}q_{\mathrm{geda},m,t}+b_{\mathrm{geda},m}$$

(24) $$q_{\mathrm{geda},m,t}-q_{\mathrm{geda},m,t-1}\leq R_{\mathrm{geu},m}$$

(25) $$q_{\mathrm{geda},m,t-1}-q_{\mathrm{geda},m,t}\leq R_{\mathrm{ged},m}$$

(26) $$q_{\mathrm{ge},m,m\mathrm{in}}\leq q_{\mathrm{geda},m,t}\leq q_{\mathrm{ge},m,\max}$$

where, *a*_geda,*m*_ and *b*_geda,*m*_ are the primary and constant term coefficients of the offer function of the *m*th thermal unit, respectively. *R*_geu,*m*_ and *R*_geu,*m*_ are the upward climbing power and downward climbing power of the *m*th thermal unit, respectively. *q*_ge,*m*,max_ and *q*_ge,*m*,min_ are the upper and lower power limits of the *m*th thermal unit, respectively.

5. Trading constraints with RL

Based on the participation of RL in trading, the current offer for RL, which is related to the subsidy and the advance notice time, is set to a constant offer before the day, and its operational constraint can be expressed as:

(27) $$0\leq q_{\mathrm{dlda},n,t}\leq q_{\mathrm{dl},n,max}$$

(28) $$\sum_{t}q_{\mathrm{dlda},n,t}\Delta t\leq q_{\mathrm{dlm},n}$$

where, *q*_dl,*n*,max_ is the upper power limit of the *n*th RL. *q*_dlm,*n*_ is the upper limit of the all-day regulated power of the *n*th RL.

### 3.3. Intra-Day Trading Clearing Model of SGLS

#### 3.3.1. Objective Function

In the intra-day time scale, the uncertainty of renewable energy output and load make a certain deviation between the actual demand and the day-ahead trading results. In order to reduce the impact of this deviation on the supply–demand balance mechanism, a 4 h trading cycle is used to divide the whole day into 6 trading cycles and 16-point intra-day trading of SGLS with curves is carried out. For renewable energy, ES, and RL declaration price, and power curves, load users only declare power curves. For trading period *R*, rolling optimization is performed based on the cost of the day-ahead trading result and the cost of the intra-day trading result in the previous period, superimposed on the cost in the trading period *R*, as shown below.

(29) $$\begin{array}{cc} \min C_{\mathrm{DI},R} & =C_{\mathrm{DA}}+C_{\mathrm{DA},R-1}\\ & +\sum_{t\in R}(\sum_{i}\rho_{\mathrm{wtdi},i,t}q_{\mathrm{wtdi},i,t}\\ & +\sum_{j}\rho_{\mathrm{pvdi},j,t}q_{\mathrm{pvdi},j,t}\\ & +\sum_{k}\rho_{\mathrm{esdi},k,t}q_{\mathrm{esdi},k,t}\\ & +\sum_{m}\rho_{\mathrm{gedi},m,t}q_{\mathrm{gedi},m,t}\\ & +\sum_{n}\rho_{\mathrm{dldi},n,t}q_{\mathrm{dldi},n,t}) \end{array}$$

where, *C*_DI,*R*_ is the trading cost as of the *R*th trading cycle in the day, *ρ*_wtdi,*i*,*t*_ is the offer price of the *i*th WT in time slot *t* of the intra-day trading cycle *R*, *q*_wtdi,*i*,*t*_ is the winning power of the *i*th WT in time slot *t* of the intra-day trading cycle *R*, *ρ*_pvdi,*j*,*t*_ is the offer price of the *j*th WT and PV in time slot *t* of the intra-day trading cycle *R*, *q*_pvdi,*j*,*t*_ is the winning power of the *j*th WT and PV in time slot *t* of the intra-day trading cycle *R*, *ρ*_esdi,*k*,*t*_ is the offer price of the *k*th offer for the *k*th ES in session *t* of the intra-day trading cycle *R*, *q*_esdi,*k*,*t*_ is the winning power for the kth ES in session *t* of the intra-day trading cycle *R*, *ρ*_gedi,*m*,*t*_ is the offer for the *m*th thermal unit in session *t* of the intra-day trading cycle *R*, *q*_gedi,*m*,*t*_ is the winning power for the *m*th thermal unit in session *t* of the intra-day trading cycle *R*, *ρ*_dldi,*n*,*t*_ is the offer for the *n*th RL in session *t* of the intra-day trading cycle *R*, *q*_dldi,*n*,*t*_ is the winning power for the *n*th RL winning power in time slot *t* within the intra-day trading cycle *R*.

#### 3.3.2. Constraint Condition

The constraints to be met by the SGLS intra-day trading are similar to those of the day-ahead trading, except that the day-ahead and intra-day trading is superimposed. The specific constraints are as follows:

1. Supply and demand balance constraints

At the intra-day time scale, the supply and demand balance constraints to be met for trading of WT, PV, ES, RL and other resources are shown below.

(30) $$\begin{array}{ll} \sum_{l}q_{\mathrm{eldi},l,t} & -\sum_{n}\left(q_{\mathrm{dlda},n,t}+q_{\mathrm{dldi},n,t}\right)\\ & =\sum_{i}\left(q_{\mathrm{wtda},i,t}+q_{\mathrm{wtdi},i,t}\right)\\ & +\sum_{j}\left(q_{\mathrm{pvda},j,t}+q_{\mathrm{pvdi},j,t}\right)\\ & +\sum_{k}\left(q_{\mathrm{esda},k,t}+q_{\mathrm{esdi},k,t}\right)\\ & +\sum_{m}\left(q_{\mathrm{geda},m,t}+q_{\mathrm{geda},m,t}\right) \end{array}$$

where, *q*_eldi,*l*,*t*_ is the load of the *l*th load user in intra-day *t* period.

2. Trading constraints on WT and PV renewables

The quotes for WT and PV are also set to constant quotes but are higher than day-ahead trading due to the shorter lead time. The power constraints satisfied are specified as follows.

(31) $$q_{\mathrm{wtda},i,t}+q_{\mathrm{wtdi},i,t}\leq p_{\mathrm{wtdi},i,t}$$

(32) $$q_{\mathrm{pvda},j,t}+q_{\mathrm{pvdi},j,t}\leq p_{\mathrm{pvdi},j,t}$$

where, *p*_wtdi,*i*,*t*_ is the predicted power of the *i*th WT in intra-day *t* period and *p*_pvdi,*i*,*t*_ is the predicted power of the *j*th WT and PV in intra-day *t* period.

3. Trading constraints on ES

The ES is set to a constant offer during the trading cycle. The offer price is higher than the day-ahead trading, and the operating constraints of the ES to be satisfied during the trading process are the same as the day-ahead trading, and the trading constraints are specified below.

(33) $$q_{\mathrm{esda},k,t}+q_{\mathrm{esdi},k,t}=p_{\mathrm{dis},k,t}$$

4. Trading constraints on thermal power units

The offer for thermal units needs to consider both day-ahead and intra-day trading components, and the trading process needs to meet its own operational constraints, as follows.

(34) $$\begin{array}{l} \rho_{\mathrm{gedi},m,t}\\ =a_{\mathrm{geda},m}(q_{\mathrm{geda},m,t}+q_{\mathrm{gedi},m,t})+b_{\mathrm{geda},m} \end{array}$$

(35) $$\begin{array}{l} \left(q_{\mathrm{geda},m,t}+q_{\mathrm{gedi},m,t}\right)\\ -(q_{\mathrm{geda},m,t-1}+q_{\mathrm{gedi},m,t-1})\leq R_{\mathrm{geu},m} \end{array}$$

(36) $$\begin{array}{l} \left(q_{\mathrm{geda},m,t-1}+q_{\mathrm{gedi},m,t-1}\right)\\ -(q_{\mathrm{geda},m,t}+q_{\mathrm{gedi},m,t})\leq R_{\mathrm{ged},m} \end{array}$$

(37) $$q_{\mathrm{ge},m,m\mathrm{in}}\leq (q_{\mathrm{geda},m,t}+q_{\mathrm{gedi},m,t})\leq q_{\mathrm{ge},m,\max}$$

5. Trading constraints for RL

The intra-day offer for the RL is set to a constant offer above the day-ahead offer, and its operational constraint can be expressed as:

(38) $$0\leq q_{\mathrm{dlda},n,t}+q_{\mathrm{dldi},n,t}\leq q_{\mathrm{dl},n,max}$$

(39) $$\sum_{t}(q_{\mathrm{dlda},n,t}+q_{\mathrm{dldi},n,t})\Delta t\leq q_{\mathrm{dlm},n}$$

### 3.4. Simulation Flow of Multi-Time Scale Trading of SGLS Based on Continuous Trading Mechanism

**Figure 3:**
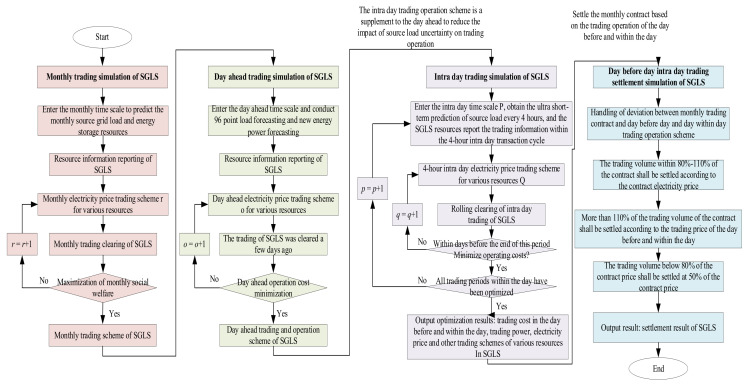
Multi-time scale trading simulation flow of SGLS based on continuous trading mechanism.

The simulation process of multi-timescale trading of SGLS based on the continuous trading mechanism proposed in this paper is shown in Figure 3. Firstly, the monthly trading simulation of SGLS is carried out. Entering the monthly time scale, the monthly prediction of SGLS resources is obtained, and the amount and quotation information are reported. The monthly electricity price trading scheme *r* of various resources is initialized. The monthly trading clearing of SGLS is carried out with the goal of maximizing social service. The clearing model is a nonlinear programming model, and CONOPT3 is used to solve the monthly trading scheme of SGLS. Secondly, the day-ahead trading simulation of SGLS is carried out. The day-ahead time scale is entered to obtain the source-load prediction data at 96 points in the region. SGLS resources completed the information reporting, and the day-ahead electricity price trading scheme of various resources is initialized. The day-ahead trading clearing of SGLS is carried out with the goal of minimizing the day-ahead operation cost. The clearing model is a nonlinear programming model, and CONOPT3 is used to solve the day-ahead trading and operation scheme of SGLS. Then, the intra-day trading simulation of SGLS is carried out. Each 4 h is an intra-day trading cycle and the intra-day time scale *p* is entered to obtain the ultra-short-term prediction data of SGLS in this trading period. The SGLS resources reports the trading information and initializes the intra-day electricity price trading scheme *q* of all kinds of resources in period *p*. The intra-day trading of SGLS is rolled out with the goal of minimizing the day-ahead intra-day operation cost as the end of this period. The clearing model is a continuous rolling nonlinear programming model. EMP and CONOPT3 are used for continuous optimization solutions until the end of all intra-day trading period optimization, and the day-ahead intra-day trading cost, the trading power, and electricity prices of all kinds of SGLS resources are obtained. Finally, the monthly–day-ahead–day trading settlement simulation of SGLS is carried out. The deviation between the monthly trading contract and the day-ahead day trading operation scheme is processed. For the trading volume that is actually implemented in the range of 80–100% of the monthly contract, the settlement is carried out according to the contract price. For the trading volume that is actually implemented in the monthly contract more than 110%, the settlement is carried out according to the day-ahead day trading price. For the trading volume that is actually implemented in the monthly contract less than 80%, the settlement is carried out according to 50% of the contract price. The settlement results of the SGLS transaction are obtained using accounting.

## 4. Case Analysis

### 4.1. Case Basic Data

In order to verify the feasibility and effectiveness of the SGLS continuous trading mechanism proposed in this paper, the simulation and optimization analysis of continuous trading in SGLS were carried out. The case analysis was performed based on the CONOPT3 solver of the GAMS platform. The constructed arithmetic case environment includes 2 wind turbines, 3 photovoltaic units, 2 energy storage units, 3 gas turbines, 3 regulated loads, and 5w load users. The charging and discharging efficiency of the energy storage was taken as 0.9 and the rest of the parameters are shown in Table 1.

**Figure 4:**
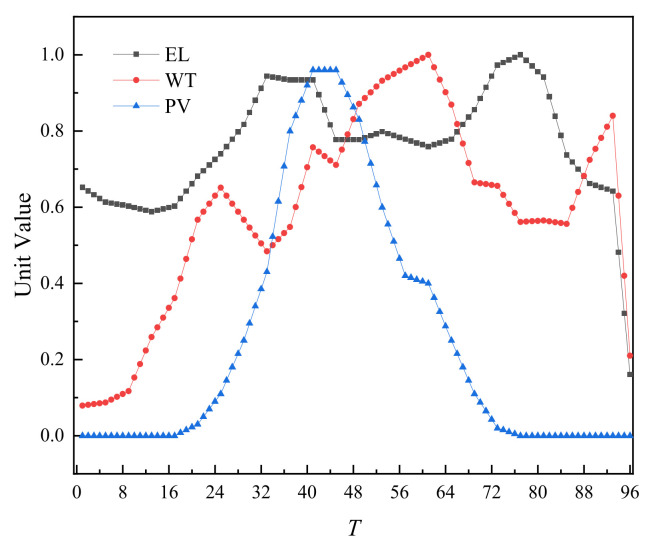
Fiducial value of load, wind power, and photovoltaic output.

The fiducial values for load, WT output, and PV output are shown in the Figure 4.

### 4.2. Result Valid Analysis

#### 4.2.1. Scenario Setting

In order to compare the effectiveness of the proposed monthly, day-ahead, and intra-day interactive trading mechanism of SGLS in this paper, two trading scenarios were set up as follows.

S1: Trading strategy of SGLS based on continuous trading mechanism.

S2: Trading strategy of SGLS based on deviation assessment.

S1 is the method proposed in this paper, which adopted the model in the previous section. S2 reduced the impact of scenery uncertainty through deviation assessment, quantified the uncertainty expression of scenery, established a deviation penalty factor and put it into the objective function of the Section 3.2 clearing model, as shown below.

(40) $$\begin{array}{ll} \min C_{\mathrm{DA}} & =\sum_{t\in T}(\sum_{i}\rho_{\mathrm{wtda},i,t}q_{\mathrm{wtda},i,t}\\ & +\sum_{j}\rho_{\mathrm{pvda},j,t}q_{\mathrm{pvda},j,t}\\ & +\sum_{k}\rho_{\mathrm{esda},k,t}q_{\mathrm{esda},k,t}\\ & +\sum_{m}\rho_{\mathrm{geda},m,t}q_{\mathrm{geda},m,t}\\ & +\sum_{n}\rho_{\mathrm{dlda},n,t}q_{\mathrm{dlda},n,t}\\ & +\sum_{i}\rho_{\mathrm{wtp},i,t}q_{\mathrm{wtp},i,t}\\ & +\sum_{j}\rho_{\mathrm{pvp},j,t}q_{\mathrm{pvp},j,t}) \end{array}$$

(41) $$q_{\mathrm{wtp},i,t}=\left\{\begin{array}{l} \Delta q_{\mathrm{wtp},i,t},\Delta q_{\mathrm{wtp},i,t}/q_{\mathrm{wtda},i,t}>5\%\\ 0,\Delta q_{\mathrm{wtp},i,t}/q_{\mathrm{wtda},i,t}\leq 5\% \end{array}\right.$$

(42) $$\Delta q_{\mathrm{wtp},i,t}=\left|q_{\mathrm{wtr},i,t}-q_{\mathrm{wtda},i,t}\right|$$

(43) $$q_{\mathrm{pvp},j,t}=\left\{\begin{array}{l} \Delta q_{\mathrm{pvp},j,t},\Delta q_{\mathrm{pvp},j,t}/q_{\mathrm{pvda},j,t}>5\%\\ 0,\Delta q_{\mathrm{pvp},j,t}/q_{\mathrm{pvda},j,t}\leq 5\% \end{array}\right.$$

(44) $$\Delta q_{\mathrm{pvp},j,t}=\left|q_{\mathrm{pvr},j,t}-q_{\mathrm{pvda},j,t}\right|$$

where, *ρ*_wtp,*i*,*t*_ is the deviation penalty price for the ith WT in day-ahead *t* period, *q*_wtp,*i*,*t*_ is the power deviation for the *i*th WT to be penalized in day-ahead *t* period, *ρ*_pvp,*j*,*t*_ is the deviation penalty price for the *j*th PV in day-ahead *t* period, *q*_pvp,*j*,*t*_ is the actual power of the ith WT in day-ahead *t* period, *q*_wtr,*i*,*t*_ is the power deviation of the *j*th PV in day-ahead *t* period, *q*_pvr,*i*,*t*_ is the actual power in day-ahead *t* period, Δ*q*_wtp,*i*,*t*_ is the power deviation of the ith WT in day-ahead *t* period, and Δ*q*_pvp,*j*,*t*_ is the power deviation of the jth PV unit in day-ahead *t* period.

#### 4.2.2. Efficiency Analysis

According to S1 and S2, using the case data in Section 4.1, the results are shown in Table 2.

According to the above Table 2, the difference between two scenarios is the day-ahead and intra-day trading sections, so the social welfare generated by monthly trading is the same. The single day-ahead trading cost of scenario S1 is CNY 960,568.23. The intra-day trading cost is CNY 3,859,967.90. The monthly settlement fee is CNY 20,264,831.76. Additionally, the consumption rate of new energy reached 100%. In scenario S2, the one-day day-ahead trading cost reached CNY 1,002,707.43. The intra-day trading cost reached CNY 4,029,300.83. The monthly settlement fee reached CNY 21,867,015.66. Additionally, the new energy consumption rate is 93.47%. Compared with the deviation assessment mechanism of S2, the interactive trading connection mechanism of monthly day-ahead intra-day SGLS under S1 scenario reduced the one-day trading cost by 4.20%, the monthly settlement cost by 7.33%, and the new energy consumption rate by 6.53%. This is mainly because the deviation assessment mechanism reduced the impact of deviation by adding the deviation penalty term to the objective function of the clearing model, and it did not increase the interactive ways of SGLS on different time scales. The comparison results verified the effectiveness of the monthly, day-ahead, and intra-day SGLS interactive trading connection mechanism in reducing trading costs and improving the consumption rate of new energy.

#### 4.2.3. Source Trading Strategy Scheme Based on Continuous Trading Mechanism

1. Monthly trading result analysis

In S1 scenario, the social welfare generated by monthly trading is CNY 7,275,990.03, and the overall trading situation is shown in Table 3.

WT and PV are traded up to the maximum forecasted volume, and the remaining resources are traded in a way that affected their own operating practices and costs, and are therefore traded in the most economical way, with monthly settlements based on this traded volume and price.

2. Day-ahead trading result analysis

**Figure 5:**
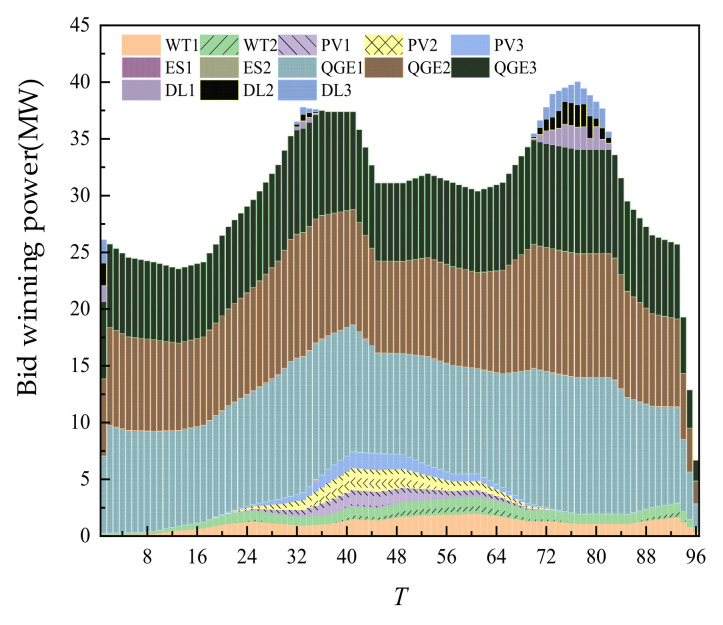
Overall trading of SGLS.

In the S1 scenario, the cost of a one-day day-ahead trading is CNY 960,568.23 and the overall trading is shown in Figure 5.

According to the Figure 5, without considering carbon emissions, most of the trading volume is provided by thermal power units. According to the characteristics of its cost curve, under a certain power threshold, its cost is lower than that of new energy, ES, and RL. Therefore, there is a considerable proportion of the trading volume of thermal power units in different periods. WT and PV are all traded in all periods, mainly because they had price advantages. ES and RL with relatively high price are mainly concentrated at 68–80 points, that is, 17–20 h. This period is the peak period of load, and the output of PV itself is 0. If thermal power continued to increase its output, its cost would rise sharply. Therefore, the load demand during this period could be met by purchasing RL power. In another load peak period, at 28–40 points, that is, 7–10 h, some RL are also traded. However, the trading volume is small, mainly because the PV had output during this period. Compared with 17–20 h, the demand for thermal power units and adjustable loads is smaller.

**Figure 6:**
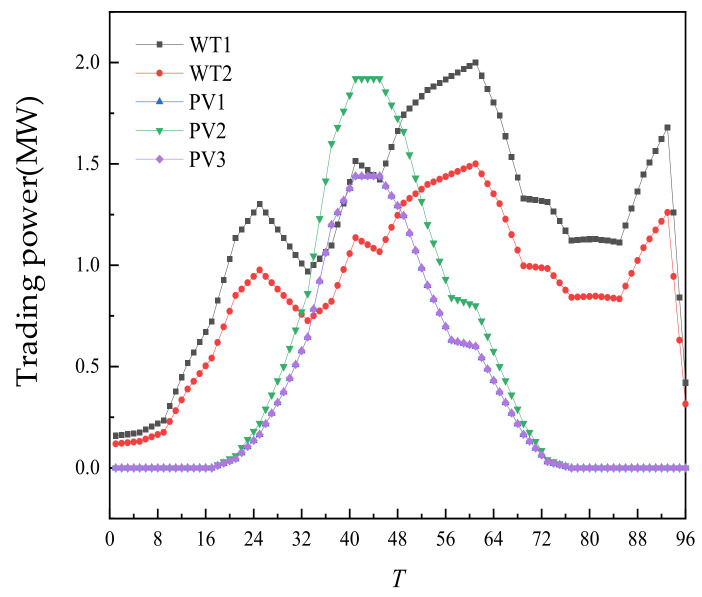
Day-ahead trading of WT and PV.

The day-ahead trading of WT and PV is shown in Figure 6. On the recent time scale, WT and PV are all traded, mainly because of their price advantages.

**Figure 7:**
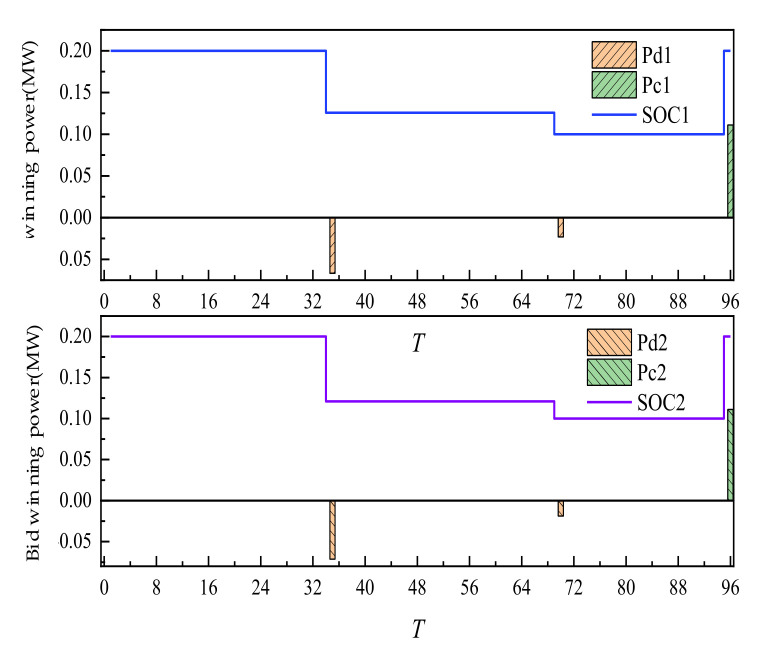
Day-ahead trading of ES.

The day-ahead trading of ES is shown in Figure 7. The positive value of the histogram represented the charging power of ES. The negative value represented the discharge power. Additionally, the curve represented the residual capacity of ES. ES participated in the trading, along with its own energy time shift process. After the trading is completed, the two ES units are in the mode of one charge and two discharges, discharging at 35 and 70 points. Additionally, charging is completed at 96 points at one time. Through energy time shift, low storage and high generation of power are realized.

**Figure 8:**
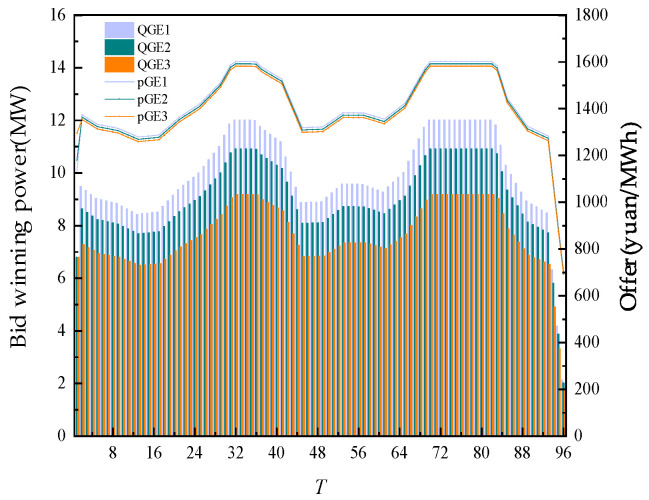
Day-ahead trading of thermal power units.

The day-ahead trading of thermal power units is shown in Figure 8 below. The output of thermal power units is closely related to their cost characteristics. The output after trading basically made the cost of 3 thermal power units at the same level. This also showed that without considering carbon emissions, from the perspective of economy, different cost characteristics of thermal power units had the most direct impact on their trading volume. At the time of trading, the unit cost of all thermal power units is almost at the same level.

**Figure 9:**
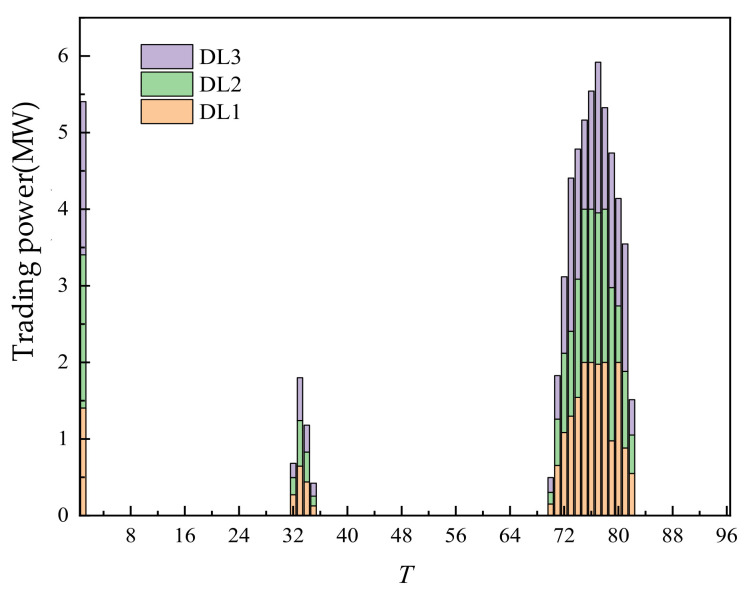
Day-ahead trading of RL.

The day-ahead trading of RL is shown in Figure 9. The trading volume of RL is mainly concentrated at points 1, 32–35, and 70–82. Combined with the load curve of the user, points 32–35 and 70–82 are peak load periods with large demand. Especially, there is no photovoltaic output at points 70–82. Increasing thermal power output would make the unit cost exceed the unit cost of RL. Therefore, RL is called to achieve supply–demand balance. The RL traded at points 32–25 is less than that at points 70–82, mainly because the PV had output during this period, and the demand for thermal power units and RL is less. The large output of RL at point 1 is mainly because there is no PV available during this period, and the trading of RL is more economical on the whole.

3. Analysis of intra-day trading results

**Figure 10:**
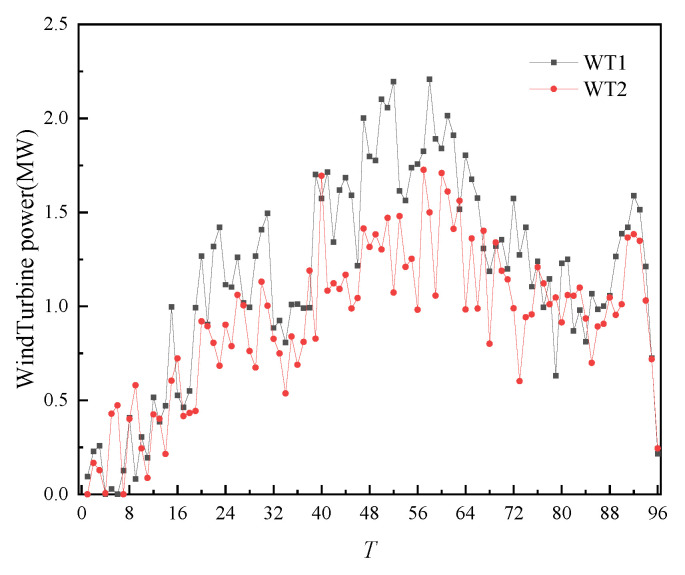
Intra-day forecast data of WT.

**Figure 11:**
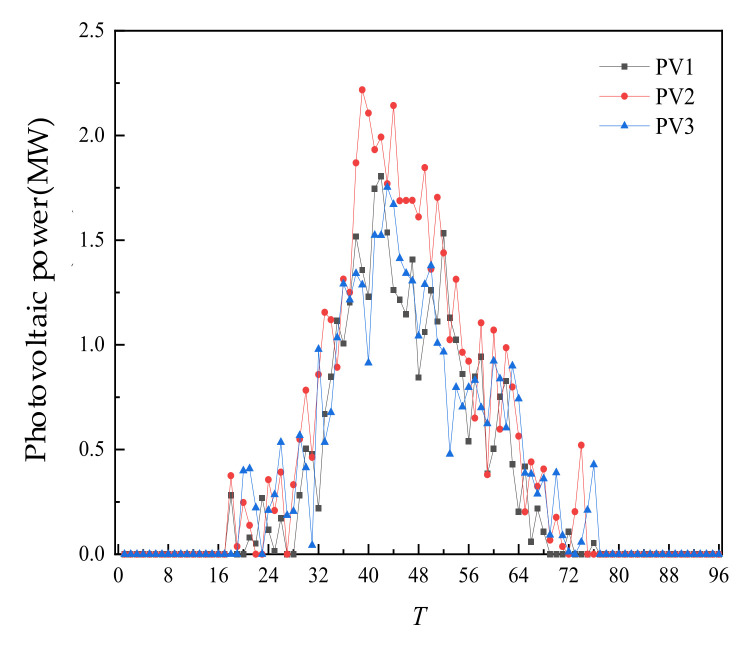
Intra-day forecast data of PV.

**Figure 12:**
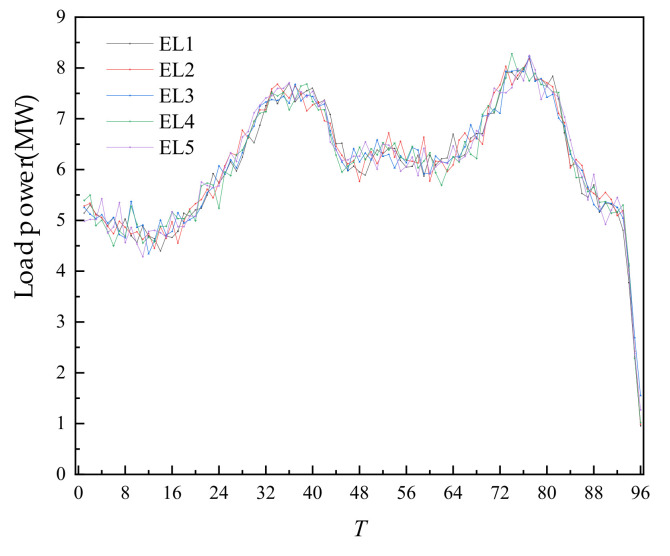
Intra-day forecast data of load.

After completing the day-ahead trading and forming a plan, the intra-day time scale was entered. In the intra-day trading of each 4 h period, the ultra-short-term power prediction and load prediction of WT and PV were carried out, and the intra-day trading was carried out according to the deviation from the day-ahead prediction. The intra-day forecast data is shown in Figure 10, Figure 11 and Figure 12 below.

In the S1 scenario, the cost of one-day trading is CNY 964,991.98. Compared with the day before trading, it had increased, due to the increase in intra-day trading volume and the fact that the quotation of market subjects’ intra-day is higher than the day-ahead.

**Figure 13:**
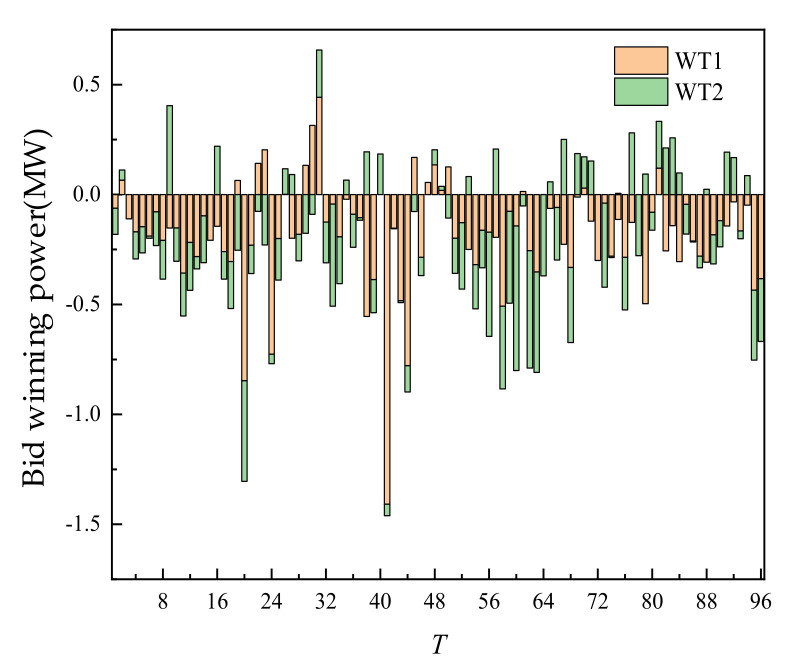
Intra-day trading of WT.

**Figure 14:**
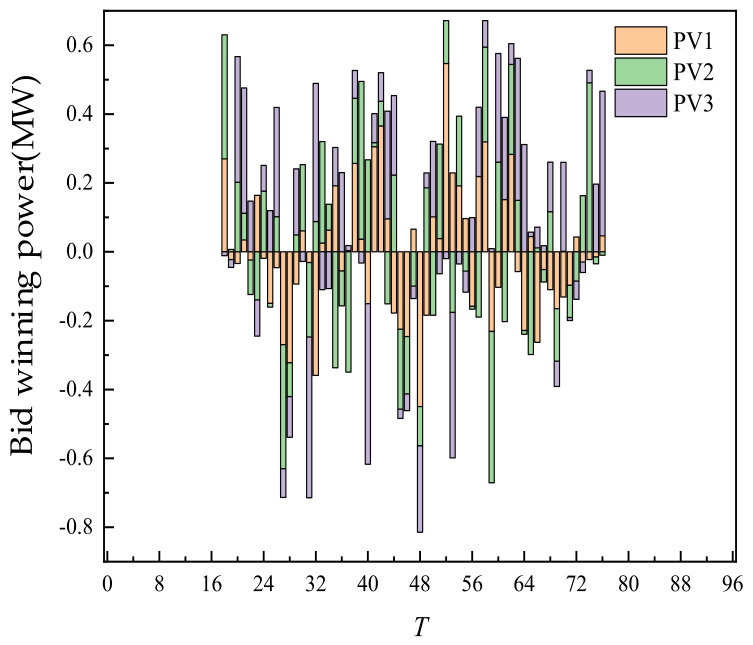
Intra-day trading of PV.

The intra-day trading of WT and PV is shown in Figure 13 and Figure 14 below. A positive value represented the power sold by WT and PV, and a negative value represented the power purchased from ES, thermal power units, and RL which is used to compensate the power part whose intra-day output does not meet the day-ahead trading volume. Compared with the day-ahead trading, the intra-day trading behaviors of new energy such as WT and PV included electricity purchase and electricity sale, which reduced the deviation between the trading value of new energy and the actual operating value from the mechanism and reduced the impact of the uncertainty of new energy output on its trading participation.

**Figure 15:**
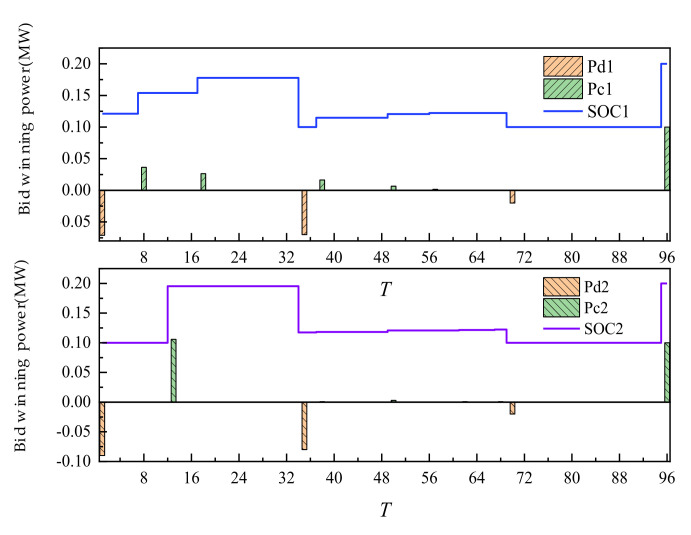
Intra-day trading of ES.

The intra-day trading of ES is shown in Figure 15 below. Compared with day-ahead trading, the amount of charging and discharging of ES increased under the condition of little change in the overall charging and discharging mode. The main reason is that the change in WT and PV output broke the original plan formulated according to day-ahead trading. It is necessary to stabilize the deviation between day-ahead trading and actual output through day-ahead trading. As a flexible adjustment resource, ES adjusted its intra-day charging and discharging period and power, participated in day-ahead trading flexibly, and reduced the adverse impact of uncertainty of new energy output.

**Figure 16:**
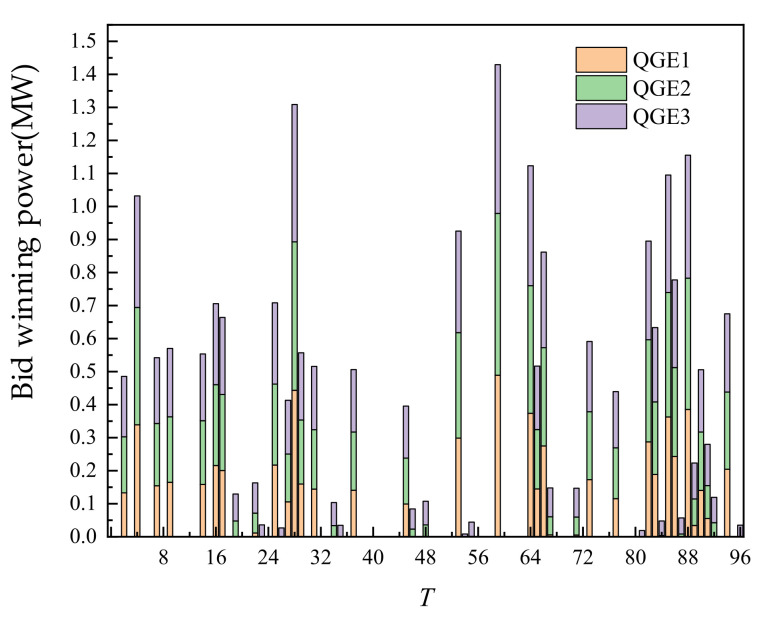
Intra-day trading of thermal power units.

The trading of thermal power units is shown in Figure 16 below. Compared with the day-ahead output increased, it is mainly to make up for the negative deviation between the operating value and the trading value caused by the reduction in WT and PV output. In the case of the increasing proportion of new energy, the load supply demand of new energy under the scenario of sudden drop in output caused by uncertainties is guaranteed.

#### 4.2.4. Sensitivity Analysis

**Figure 17:**
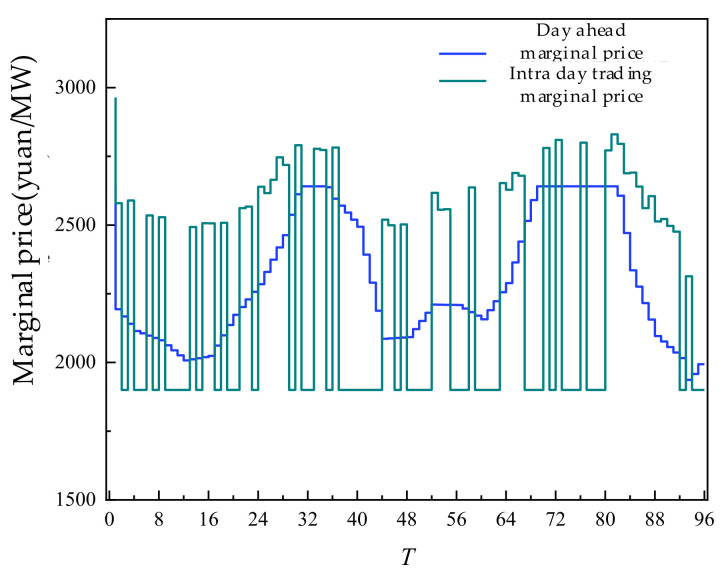
Marginal price of monthly, day-ahead, intra-day trading in SGLS.

The sensitivity at different trading times is analyzed by the marginal price of the day-ahead and intra-day trading in SGLS, as shown in Figure 17 below.

According to the comparison of monthly trading, day-ahead trading marginal price, and intra-day trading marginal price, the price fluctuation of intra-day trading is larger than that of day-ahead and monthly, and many periods reached the price boundary. Monthly trading is electricity trading, and its marginal price is a constant value. The price of intra-day trading is higher or lower than that of day-ahead trading, which reflected that in a shorter trading period, the attribute of electricity as a commodity is more obvious, and the price is determined by supply and demand. Combined with ultra-short-term load forecast and power generation forecast, according to the intra-day trading situation, it could be seen that the intra-day price is higher than the day-ahead price, mainly in the period of insufficient new energy output, and it is necessary to purchase electricity with higher price from thermal power units, resulting in higher marginal price in this period. On the contrary, the period of intra-day price lower than the day-ahead price is mostly the period of abundant new energy output. New energy sold more electricity, reduced the output of thermal power units, thereby reduced the total cost, and the marginal price of the period is accordingly reduced.

According to the comparison of trading schemes at different times in the trading strategy scheme of SGLS in Section 4.2.3, the sensitivity between trading scenarios formed at different trading times was analyzed.

Taking the 12 o’clock as an example, according to the ultra-short-term prediction of intra-day new energy and load, new energy occurred in this period, which exceeded the day-ahead prediction value. It could be seen that compared with the day-ahead planned stage of supply exceeding demand, the trading scenario at this time could be named as the new energy consumption scenario of new energy supply exceeding demand. Compared with the day-ahead trading, in this period, WT would be sold beyond the day-ahead planned WT (as shown in Figure 13). ES bought new energy by charging (as shown in Figure 15). Thermal power units had no output at this time, which achieved the effect of absorbing new energy. Taking the time 1 o’clock as an example, contrary to the time at 12 o’clock, there is less new energy at this time, which is lower than the day-ahead forecast value. It could be seen that compared with the day-ahead plan, this period is the stage of supply less than demand, and the trading scenario at this time could be named as the anti-peaking scenario of new energy with supply less than demand. Compared with the day-ahead trading, the intra-day trading of this period, and the energy storage discharge (as shown in Figure 15) could make up for the shortage of new energy output. It could be seen that the trading of SGLS based on the continuous trading mechanism could flexibly switch the trading scenarios of new energy consumption and new energy counter-peak according to the deviation between the ultra-short term source load forecast in the intra-day and the day-ahead forecast, which improved the trading efficiency and promoted the efficient utilization of new energy.

## 5. Conclusions

This paper constructed an interactive trading connection mechanism of monthly day-ahead intra-day in SGLS and constructed the trading clearing model of SGLS based on the continuous trading mechanism. Through example analysis, the main conclusions are as follows:

(1) The interactive trading connection mechanism of monthly, day-ahead, intra-day in SGLS connects the monthly trading information, day-ahead trading information, day-ahead operation plan, intra-day trading information and operation mode, which greatly reduces the impact of trading and operation deviation caused by the uncertainty of new energy output on power trading.
(2) The trading of SGLS based on the continuous trading mechanism is conducive to reducing the trading cost. Compared with the trading of SGLS based on deviation assessment, the single day trading cost is reduced by 4.20% and the new energy consumption rate is increased by 6.53%.
(3) The two trading scenarios of new energy consumption and new energy reverse peak shaving caused by the uncertainty of source load prediction were analyzed. The trading clearing model of SGLS based on continuous trading mechanism can effectively deal with the switching of intra-day two trading scenarios.
(4) Compared with the day-ahead trading and monthly trading, the intra-day trading marginal price under the continuous trading mechanism of monthly day-ahead intra-day in SGLS has greater volatility, which not only brings opportunities to the competition of market subjects, but also increases the difficulty of daily price prediction.

## Figures and Tables

**Table 1:** Basic data of the calculation example.

Trading Subject	Capacity (MW)	Monthly Quotation (CNY/MWh)	Day-Ahead Quotation (10^3^ CNY/MWh)	Intra-Day Quotation (10^3^ CNY/MWh)
WT1	2.0	*a*_wtm,1_ = 0.2315, *b*_wtm,1_ = 250.5	500	700
WT2	1.5	*a*_wtm,2_ = 0.2315, *b*_wtm,2_ = 250.5	500	700
PV1	1.5	*a*_pvm,1_ = 0.5435, *b*_pvm,1_ = 148.9	400	600
PV2	2.0	*a*_pvm,2_ = 0.5435, *b*_pvm,2_ = 148.9	400	600
PV3	1.5	*a*_pvm,3_ = 0.5435, *b*_pvm,3_ = 148.9	400	600
ES1	1.0	*a*_esm,1_ = 71.43, *b*_esm,1_ = 457.1	700	900
ES2	1.2	*a*_esm,2_ = 71.43, *b*_esm,2_ = 457.1	700	900
GT1	16.0	*a*_gem,1_ = 0.1389, *b*_gem,1_ = 450	*a*_geda,1_ = 90, *b*_geda,1_ = 520	
GT2	16.0	*a*_gem,2_ = 0.1389, *b*_gem,2_ = 450	*a*_geda,2_ = 100, *b*_geda,2_ = 500	
GT3	16.0	*a*_gem,2_ = 0.1389, *b*_gem,2_ = 450	*a*_geda,3_ = 120, *b*_geda,3_ = 480	
DL1	2.0	*a*_dlm,1_ = 3.333, *b*_gem,1_ = 250	800	1000
DL2	2.0	*a*_dlm,2_ = 3.333, *b*_gem,2_ = 250	800	1000
DL3	2.0	*a*_dlm,3_ = 3.333, *b*_gem,3_ = 250	800	1000
EL1	8.0	*a*_elm,1_ = −10, *b*_elm,1_ = 44,900	/	/
EL2	8.0	*a*_elm,2_ = −10, *b*_elm,2_ = 44,900	/	/
EL3	8.0	*a*_elm,3_ = −10, *b*_elm,3_ = 44,900	/	/
EL4	8.0	*a*_elm,4_ = −10, *b*_elm,4_ = 44,900	/	/
EL5	8.0	*a*_elm,5_ = −10, *b*_elm,5_ = 44,900	/	/

**Table 2:** Trading results in different scenarios.

Scenario	Monthly Trading Social Welfare (CNY)	Day-Ahead Trading Cost (CNY)	Single Day Trading Cost (CNY)	Monthly Settlement Expenses (CNY)	New Energy Consumption Rate (%)
S1	7,275,990.03	960,568.23	964,991.98	20,264,831.76	100.00
S2	7,275,990.03	1,002,707.43	1,007,325.21	21,867,015.66	93.47

**Table 3:** Monthly trading.

Trading Objects	Electricity Turnover (MWh)	Sold Price (CNY/MWh)
WT1	861.86	450.02
WT2	646.40	400.14
PV1	277.65	299.80
PV2	370.20	350.10
PV3	277.65	299.80
ES1	12.06	1318.64
ES2	12.06	1318.64
GT1	6228.15	1315.09
GT2	6228.15	1315.09
GT3	6228.15	1315.09
DL1	289.56	1215.09
DL2	289.56	1215.09
DL3	289.56	1215.09
